# Pre-Referral Microbiology in Long Bone Infection: What Can It Tell Us?

**DOI:** 10.3390/antibiotics12010013

**Published:** 2022-12-22

**Authors:** Andrew J. Hotchen, Ruth A. Corrigan, Maria Dudareva, Andrew Bernard, Jamie Ferguson, Bridget L. Atkins, Martin McNally

**Affiliations:** 1 Bone Infection Unit, Nuffield Orthopaedic Centre, Oxford University Hospitals, Oxford OX3 7LD, UK; 2 Nuffield Division of Clinical Laboratory Sciences, University of Oxford, John Radcliffe Hospital, Headley Way, Oxford OX3 9DU, UK

**Keywords:** osteomyelitis, fracture-related infection, diagnosis, pre-referral microbiology, microbiological sampling

## Abstract

*Background*: It remains unclear how accurately patients’ previous microbiology correlates with that ascertained from deep sampling in long bone infection. This study assessed the quality of microbiology referral information and compared it to the gold standard of intra-operative deep tissue sampling. *Methods*: All patients referred to a single specialist centre within the UK between January 2019 and March 2020 who received surgery for long bone infection were eligible for inclusion. Data on microbiological testing that was performed prior to referral was collected prospectively at the time of clinic appointment and prior to surgery. Pre-referral microbiology was compared to microbiology from deep tissue samples taken during surgery. *Results*: 141 patients met the diagnostic criteria for long bone infection and were included for analysis. Of these, 72 patients had microbiological information available at referral from 88 samples, obtained from either sinus swab (n = 40), previous surgical sampling (n = 25), biopsy (n = 19) or blood cultures (n = 4). In 65.9% of samples, pre-referral microbiology was deemed to be a non-match when compared to intra-operative samples. Factors that increased risk of a non-match included presence of a sinus (odd’s ratio (OR) 11.3 [95% CI 2.84–56.6], *p* = 0.001), increased duration of time from sampling (OR 2.29, [95% CI 1.23–5.90], *p* = 0.030) and results from prior surgical sampling (OR 23.0 [95% CI 2.80–525.6], *p* = 0.011). Furthermore, previous surgical debridement gave an increased risk of multi-, extensively or pan-resistant isolates cultured from intra-operative sampling (OR 3.6 [95% CI 1.5–8.7], *p* < 0.01). *Conclusions*: We have demonstrated that presence of a sinus, a long time from the sample being taken and results from prior surgical sampling are more likely to give inaccurate representation of current microbiology. Importantly, in cases with previous debridement surgery, there was an increased risk of multi drug resistant isolates which should be planned for in future treatments.

## 1. Introduction

Bone and joint infections can be complex, requiring multi-disciplinary care for optimal management [1,2,3] and are associated with lower self-reported quality of life than that of the general population [4,5]. Complex cases may benefit from early referral to specialist centres to facilitate access to a multi-disciplinary team which includes orthopaedic and plastic surgeons, infectious disease physicians/microbiologists, histopathologists, musculoskeletal radiologists, physiotherapists, and specialist nurses. Although all members of this team are important, infection physicians who optimise patients’ medical co-morbidities and determine bespoke antibiotic regimes are considered particularly helpful [2,6,7].

The gold standard sampling method for microbiological culture is intra-operative deep tissue sampling, taken when a patient has not been taking antibiotics for at least 2 weeks. During sampling, a minimum of five microbiological samples taken with separate instruments have been shown to be optimal [8,9]. Identical growth of species and antibiogram from at least 2 samples is indicative of infection and can be used to guide antibiotic therapy [9,10]. Although the results of a majority of cultures are available by day 3 [11], certain isolates may take longer, and in 30–40% of cases, cultures remain negative, despite the presence of other features diagnostic of infection (for example, a sinus, or histology positive for infection) [9].

Patients with complex bone and joint infections are commonly referred to more than one centre for treatment [5,12]. Therefore, at the time of referral to a specialist bone infection unit, many patients will have already undergone previous surgical procedures with microbiological sampling and received multiple courses of antibiotics. It is hypothesised that resistance of isolated pathogens is likely to increase with the number of previous surgical procedures and/or antibiotic courses [13]. Furthermore, antimicrobial options in patients with multi- drug resistant isolates are limited, warranting management by a specialist infectious diseases physician. This forms the basis of the ‘antimicrobial options’ variable of the BACH and subsequently the JS-BACH (Joint-specific; Bone involvement; Antimicrobial options; Coverage of the soft tissues; Host status) classification system of bone and joint infection. The JS-BACH classification evolved from the BACH classification to include prosthetic joint infection in addition to long bone osteomyelitis [5]. Both of which demonstrated that antimicrobial profile can predict outcomes after surgery [4,5].

Previous microbiology and recent antibiotic use are considered by infectious disease physicians when making antimicrobial therapy plans [14]. This is applicable to both empiric regimens, particularly in cases of multi, extensively or pan drug resistant isolates (MDR, XDR or PDR, respectively) and targeted regimes. Prior microbiological cultures may be the only available information when the patient has received antibiotics in the two weeks prior to sampling. However, often previous microbiological sampling is inadequate, for example from superficial wound or sinus swabs [15].

It remains unclear how accurately patients’ previous microbiology correlates with that ascertained from deep sampling, at definitive surgery in a specialist centre. Indeed, knowledge of whether patients possess risk factors for highly resistant isolates pre-operatively may help prompt early referral to a specialist centre. This study assessed the quality of microbiology referral information and compared it to the gold standard of intra-operative deep tissue sampling.

## 2. Results

### 2.1. Demographics and Sampling Information

**Figure 1:**
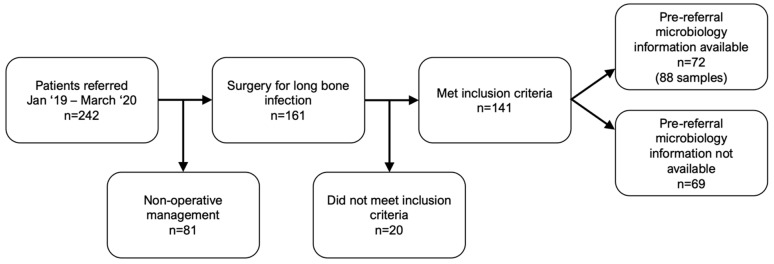
Flow diagram of patient inclusion in the study.

A total of 242 patients were referred with suspected bone infection between January 2019 and March 2020. Of these, 161 had subsequent surgery and 141 met our diagnostic criteria for infection and were included for analysis (Figure 1). 41 (29.1%) of these patients had already undergone surgical debridement prior to referral. Patient demographic data are shown in Table 1. Of the 141 patients who received surgery, information on microbiology sampling prior to referral was unavailable in 69 (48.9%). These were classified as Ax according to the anti-microbial options of the JS-BACH classification. The patients with microbiology information available from referral were classified as either Ax/A1 (n = 62) or A2/A3 (n = 10).

**Figure 2:**
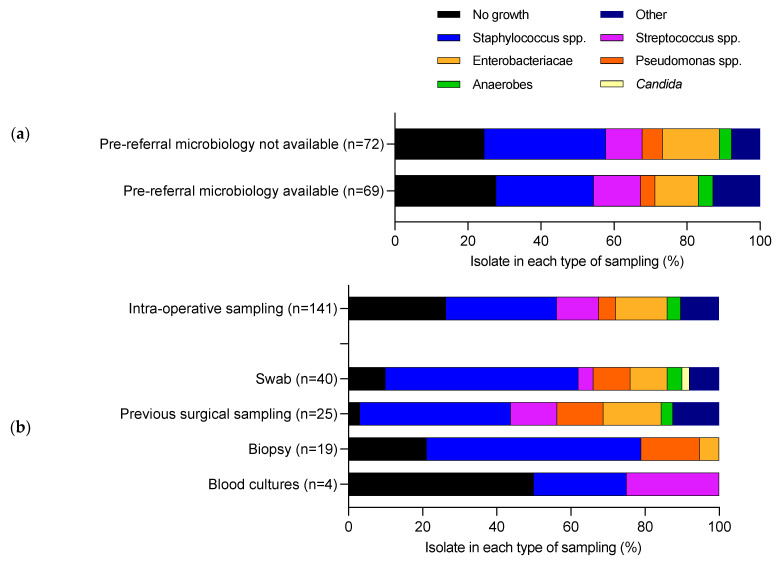
Comparison of the microbiology from intra-operative sampling versus microbiology from before referral. (**a**) Comparison of intra-operative sampling from patients who had pre-referral microbiology available and not available. (**b**) Distribution of each species isolated from each of the different types of sampling method that was used on pre-referral microbiology versus intra-operative sampling.

Seventy-two patients had microbiology data available at the time of referral, which was derived from 88 microbiology samples. These were comprised of wound or sinus swabs (40/88, 45.5%), previous surgical sampling (25/88, 28.4%), biopsy (19/88, 21.6%) or blood culture (4/88, 4.5%), Table 1. The median interval between date of referral microbiology and intra-operative sampling was 0.8 years (Inter-quartile range 0.49–1.53 years) and 26.1% (23/88) were within six months of the surgical sampling date. Pre-referral microbiology was predominantly comprised of *Staphylococcus* spp. (31/88, 35.2%), which was the most common species in each sampling method (Figure 2a). There was similar intra-operative microbiological growth in patients who were referred with microbiology available and those referred without microbiology available (Figure 2b).

### 2.2. Pre-Referral Microbiology Versus Intra-Operative Sampling

In 25.0% (22/88) of samples, all isolates identified before referral were also identified during intra-operative sampling and deemed to be a ‘complete match’ (Table 2). A further 9.1% (8/88) were deemed to be a ‘partial match’ where all isolates from pre-referral microbiology were confirmed, with additional isolates identified from intra-operative sampling. The remaining 65.9% (58/88) had pre-referral microbiology that was different when compared with intra-operative samples and deemed to be a ‘non-match’ (Table 2).

### 2.3. Factors Predicting Different Pre-Referral Microbiology from Intra-Operative Sampling Isolates

**Figure 3:**
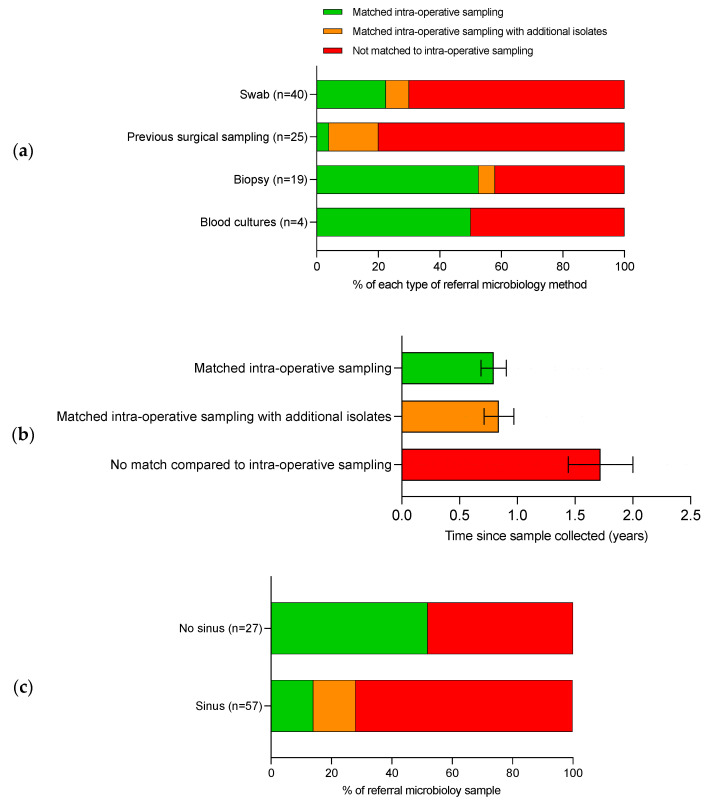
Outcomes of pre-referral microbiology compared to intra-operative sampling. Comparison of pre-referral microbiology to intra-operative sampling by (**a**) method of sampling, (**b**) timing from sampling to operative samples and (**c**) the presence of an active sinus. ‘Complete matches’ are in green, ‘partial matches’ are in orange and ‘non-matches’ are in red.

Patient factors hypothesised to increase the chance of a ‘partial match’ or ‘non-match’ were: increased time between pre-referral microbiology and intra-operative sampling and the presence of a sinus (Figure 3a–c). Previous surgical sampling gave the lowest number of ‘complete matches’ to intra-operative sampling at 4.0% (1/25) (Figure 3a). Increased time from the pre-referral microbiology sample was significantly associated with a ‘non-match’ on intra-operative sampling (*p* < 0.05, simple logistic regression) (Figure 3b). Patients with a sinus had pre-referral microbiology that did not match intra-operative sampling in 86.9% which compared to 48.2% in patients who did not have a sinus (Figure 3c).

**Figure 4:**
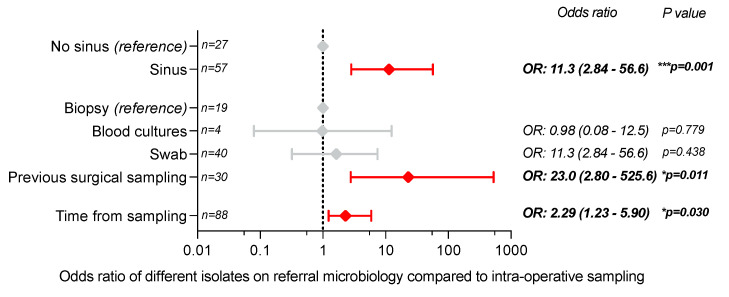
Multivariate logistic regression demonstrating that the presence of an active sinus, isolates from previous surgical sampling and increased time interval between referral sample and intra-operative sample all independently predicted an incorrect match of referral microbiology to intra-operative samples. * *p* < 0.05, and *** *p* < 0.001. OR = Odds ratio.

To assess for potential confounders, a multivariate logistic regression model was built to assess whether referral microbiology type, duration from pre-referral microbiology to intro-operative sampling and the presence of a sinus were independent predictors of a discrepancy between referral microbiology and microbiology obtained from intra-operative sampling (Nagelkerke pseudo-R^2^ = 0.479; Hosmer-Lemeshow statistic 10.0, *p* = 0.263). Presence of a sinus (OR) 11.3 [95% CI 2.84–56.6], *p* = 0.001), increased duration of time from sampling (OR 2.29, [95% CI 1.23–5.90], *p* = 0.030) and method of pre-referral microbiology being previous surgical sampling (OR 23.0 [95% CI 2.80–525.6], *p* = 0.011) were independent predictors of incorrect matching of samples to intra-operative specimens (Figure 4).

### 2.4. Factors Predicting Increased Resistance Patterns on Intra-Operative Sampling

Patients were significantly less likely to have microbiology isolates classified as A2 (MDR or equivalent) or A3 (PDR and XDR) from pre-referral microbiology samples (10/72, 13.9%) compared to samples taken at intra-operative sampling (19/72, 22.6%; *p* = 0.030 two-tailed paired binomial test). The proportion of patients classified as A2 or A3 following intra-operative sampling were similar in the group where pre-referral microbiology was unavailable compared to the group where pre-referral microbiology was available (15.9% vs. 23.6%, *p* = 0.299, two-tailed Fisher’s exact test).

To assess factors that were able to predict the presence of increased resistance patterns on intra-operative sampling, a multivariate model was built (Nagelkerke pseudo-R^2^ = 0.10; Hosmer-Lemeshow statistic 9.5, *p* = 0.146). Controlling for the classification of the microbiology received at referral, previous surgical debridement was significantly associated with an intra-operative sampling classification of A2 or A3 (OR 3.6 [95% CI 1.5–8.7], *p* < 0.01) (Table 3).

## 3. Discussion

This study has presented the pre-referral microbiology for a series of 141 patients who received surgery for long bone infection in a specialist centre. At the time of referral, 51.1% of patients had pre-referral information available but only 25% of these samples gave a complete match to samples taken at the time of operation. This highlights the need for up to date diagnostic sampling in all surgery for suspected bone infection.

Our series has demonstrated the wide range of sampling methods used to identify pathogens. Single superficial wound swabs were common despite published guidance advising against this type of investigation [16,17,18]. There were major differences in the concordance between methods and intra-operative sampling, but no pre-referral method was reliable. Previous biopsy was the most accurate but did not reach 60% concordance.

Previous studies have compared isolates obtained from sinus swabs to those obtained from intra-operative sampling with varying results. Bernard et al., demonstrated that two separate identical sinus samples had a 96% association with isolates grown at the time of intra-operative sampling [19]. Other studies have reported lower correlation between sinus tract culture and intra-operative sampling in osteomyelitis at 38% to 67% [20,21,22]. However, isolation of *Staphylococcus aureus* on sinus swab correlated with intra-operative sampling [23]. More recently, Tawfik et al., presented a systematic review of 6 studies including 281 patients which demonstrated that only 47% of patients who had isolation of *Staphylococcus aureus* from sinus swabs also had isolation of *Staphylococcus aureus* from intra-operative specimens [15]. Other *Staphylococcus* isolates had an 8% concordance and *Streptococci* had a 22% concordance between sinus swab and intra-operative specimens. Other bacteria were presented as having a low concordance between non-bone microbiology and intra-operative sampling at <26% [15].

The time from initial pre-referral microbiology to definitive intra-operative sampling was long (median 0.8 years). However, it has been shown, in fracture-related infection that time alone does not determine the microbiological diagnosis [24,25,26,27]. The differences observed between pre-referral microbiology and intra-operative sampling may be secondary to patients having received (i) multiple courses of antibiotics, (ii) the changing microbiology that surrounds a sinus or open wound, and (iii) the selective antimicrobial treatment of isolates from previous surgeries. Clearly each of these issues will become more likely as time progresses from the initial diagnostic testing. When considering these mechanisms, it is important to distinguish between cases with a ‘closed system infection’ (no open wound, sinus or open surgery) and those where the infected area in the bone communicates with the external environment. In a closed infection, there are very limited means by which the pathogens can change. Antibiograms of infecting organisms can be altered by exposure to systemic antimicrobial therapy but new species can only arise from haematogenous spread, which is unlikely. When a sinus or wound is present, or after unsuccessful open surgery, exchange of organisms or ingress of additional pathogens is possible. Treatment with antimicrobials may select certain species or promote altered resistance profiles.

The presence of a sinus, increased duration from pre-referral sampling to intra-operative sampling and microbiology taken from previous surgery all gave an increased risk of non-concordance with definitive microbiological cultures. The presence of a sinus and microbiology from previous surgeries increased the risk of non-concordance by 11.3 times and 23.0 times, respectively. For every year increase between previous microbiology and index microbiology (taken at surgery), the risk of non-concordance increases by 2.3 times. Furthermore, having previous bone debridement for bone infection increased the risk of multi-drug resistance (MDR), pan drug resistant (PDR) or extensively drug resistant (XDR) isolates being isolated at intra-operative sampling. These data suggest that pre-referral microbiology should be interpreted with caution, and that patients who have had multiple previous treatments should be deemed high risk for the presence of MDR, XDR, or PDR isolates. It has been previously demonstrated that this adversely affects outcome for treatment of long bone infection [5].

The main difficulty in decision-making around antibiotic therapy arises when intraoperative sampling does not produce a positive microbial culture but other features (sinus drainage, histology, imaging) all suggest infection. This can affect up to 30% of patients, mainly due to the use of antibiotics at the time of surgical sampling [24]. In these cases, it is tempting to use previous microbiological cultures to direct treatment. These data suggest that this would not be an appropriate strategy. If the pre-referral microbiology was recent and obtained from a closed system infection, it may be more valid but still has a fairly high non-concordance risk. We would advise that repeat sampling should be attempted, off antimicrobials (if possible) or locally derived empiric antimicrobial policies should be followed [7]. These should be based on an assessment of the likely local bacterial flora, encountered in the region [14].

There are limitations to this study. This study included prospective and retrospective data collection. Retrospective data included whether patients had ‘previous debridement’ surgery. ‘Previous debridement’ surgery was limited to either excision of bone, or reaming of the intra-medullary canal. This information was gained, where possible, from the notes, but in some cases relied on patient recollection of events, which can be unreliable. This study did not quantify the increase in risk of MDR, XDR or PDR isolates per previous procedure due to inadequate power and the quality of retrospective data. Information about pre-referral microbiology was recorded from previous clinic notes or referral information. There is a possibility that some patients who had pre-referral microbiology performed were not referred with that information, particularly when they did not grow any isolate. In addition, the methodology of sampling technique between patients could have varied and we do not have data pertaining to antibiotic administration prior to sampling. Another factor that may have caused discrepancy between isolates grown from pre-referral microbiology compared to intra-operative samples was that samples were analysed in different hospitals. Different hospitals have varying protocols used to culture bacteria, which could yield differing results.

We have presented a series of 141 cases who have been referred to a specialist bone infection centre within the UK. Pre-referral microbiology may be useful in cases where surgery is not being performed and antibiotic suppression is required, or in cases where there is no growth during intra-operative sampling. We have demonstrated that the presence of a sinus, a long time from the sample being taken and the results from surgical sampling are more likely to give inaccurate representation of current microbiology. Importantly, in the cases of previous debridement surgery, there is an increased risk in having either MDR, XDR or PDR isolates which should be planned for in future treatments.

## 4. Materials and Methods

### 4.1. Patient Identification

Patients with long bone infection (including osteomyelitis and fracture related infections) were identified at a single specialist referral centre for bone and joint infection (Oxford University Hospitals, Oxford, UK). Patients referred between January 2019 and March 2020 were identified prospectively at clinic. Patients who subsequently underwent surgery were assessed for a confirmed diagnosis of infection. Patients were included if they had any of the following at the time of surgery: (i) positive microbiological culture (confirmed with indistinguishable organisms cultured from two or more deep tissue specimens), (ii) histology confirming active infection or (iii) pus or sinus present as defined in previous studies [16,28].

### 4.2. Data Acquisition

Data was collected prospectively on all patients who attended clinic for suspected long bone infection. Data collected included the availability of microbiology and, if available, the date of microbiology, sampling method, isolate and results of susceptibility testing. Patient details included presence of a draining sinus, site of the infection and whether the patient had undergone a previous bone debridement. Classification according to the ‘antimicrobial options’ of the JS-BACH classification system was performed at the time of clinic, based on the available microbiology [4]. For those patients who subsequently underwent surgery, data was collected regarding the date of surgery, pathogens identified, pathogen antibiogram and the resultant JS-BACH classification. Classification was confirmed retrospectively by a member of the team who was not directly involved in clinical care of the patients in a blinded manner. Any discrepancy in classification was resolved by the senior author.

### 4.3. Comparison of Pre-Referral Microbiology to Intra-Operative Sampling

Microbiology received at the time of referral was compared to the microbiology obtained from intra-operative sampling both in terms of pathogen species and its antibiogram. For patients who were referred with microbiology from more than one operation or procedure, the comparison between pre-referral and intra-operative microbiology was performed per pre-referral sample.

Pre-referral microbiology was deemed a ‘complete match’ if (i) species and antibiogram between pre-referral microbiology and intra-operative sampling were identical (on available susceptibility testing), or (ii) there was no growth on both intra-operative sampling and pre-referral microbiology. Pre-referral microbiology was deemed a ‘partial match’ if there was a complete match of species and antibiogram between pre-referral microbiology and intra-operative sampling but with additional isolates cultured at the time of intra-operative sampling. Pre-referral microbiology was deemed to be a ‘non-match’ if: (i) any isolate identified during pre-referral microbiology was not present on intra-operative sampling, (ii) pre-referral microbiology was culture positive and intra-operative samples were culture negative, (iii) pre-referral microbiology was culture negative and intra-operative sampling was culture positive.

### 4.4. Statistical Analysis

Continuous variables were reported using means and standard deviations (SD), if normally distributed and medians and inter-quartile ranges (IQR) if non-normally distributed. Categorical variables were presented as percentages with 95% confidence intervals (CIs).

Pre-referral microbiology was compared to samples obtained intra-operatively and reported per pre-referral microbiology sample. To assess factors where intra-operative sampling was a ‘non-match’ to pre-referral microbiology, a multivariate logistic regression was performed. Variables hypothesised to influence this included (i) presence of a draining sinus, (ii) time from pre-referral microbiology sampling and (iii) pre-referral sampling methodology.

To assess whether factors that predicted a discrepancy between factors hypothesized to increase the risk of a discrepancy between pre-referral microbiology and intra-operative sampling, a multi-variate regression model was built. Factors included in the model were (i) the presence of a sinus, (ii) increased time from pre-referral microbiology and intra-operative sampling and (iii) the pre-referral microbiology sampling method.

To assess whether patient factors available at referral were able to predict the presence of MDR, XDR or PDR isolates (JS-BACH classification A2 or A3) on intra-operative sampling, a multivariate logistic regression model was built. Variables were included based on the hypothesis that they would influence the presence of resistant isolates from intra-operative sampling. These included whether the patient had received previous bone debridement and patients who had MDR (JS-BACH A2), PDR or XDR (JS-BACH A3) isolates from pre-referral microbiology.

For all statistical tests, a significance level of <0.05 was used. Statistical analyses were performed using R in RStudio (version 4.0.2, 22 June 2020, Boston, MA, USA).

## Figures and Tables

**Table 1:** Demographics, referral, and microbiological information of the 141 patients included.

Age (Mean, SD)		Mean 51.2 Years (SD 17.3 Years)
Site of infection		
	Upper limb	30
	Lower limb	111
Aetiology of infection		
	Fracture related infection – closed	45 (31.9%)
	Fracture related infection – open	40 (28.4%)
	Haematogenous	26 (18.4%)
	Contiguous focus	15 (10.6%)
	Post-orthopaedic procedure (non-fracture)	15 (10.6%)
Pre-referral microbiology not available		69
Pre-referral microbiology available (n = 88) *		
	Swab	40
	Previous surgical sampling	25
	Biopsy	19
	Blood culture	4
Time from pre-referral microbiology to surgery (n = 88) *		median 0.8 years (IQR 0.49–1.53 years)
JS-BACH Classification of pre-referral microbiology		
	No referral information (Ax)	69
	Ax/A1	62
	A2/A3 (MDR+)	10
JS-BACH Classification from intra-operative sampling		
	Ax/A1	113
	A2/A3 (MDR+)	28
Previous debridement for osteomyelitis		
	Yes	41
	No	100

* 7 patients had multiple methods of pre-referral microbiology.

**Table 2:** Comparison of pre-referral microbiology to intra-operative samples in 72 patients representing a total of 88 pre-referral microbiology samples.

Growth from Referral Microbiology Samples		Growth Reported from Intra-Operative Sampling	Number
‘Complete match’			
	Yes	Same as referral microbiology	16 (18.2%)
	No	No growth	6 (6.8%)
‘Partial match’			
	Yes	Same as referral microbiology with additional isolates	8 (9.1%)
‘Non-match’			
	No	Significant growth	6 (6.8%)
	Yes	Isolates not present on referral microbiology samples	52 (59.1%)

**Table 3:** Results from multivariate logistic regression assessing MDR+ isolates (A2/A3) versus non-MDR isolates (Ax/A1) during intra-operative sampling. Previous bone debridement of osteomyelitis increased odds of A2/A3 isolate at intra-operative sampling by 3.6 (1.5–8.7), ** *p* < 0.01.

			Intra-Operative Classification			
		Total	Ax/A1	A2/A3 (MDR+)	Odds Ratio	95% CI
Referral microbiology						
	Not available (Ax)	69	58 (84.1%)	11 (15.9%)	-	
	Ax/A1	61	47 (77.1%)	14 (22.9%)	1.2	0.44–3.2
	A2/A3	11	8 (72.7%)	3 (27.3%)	1.4	0.26–5.6
Previous debridement						
	No (reference)	99	84 (84.8%)	15 (15.2%)	-	
	Yes	42	29 (69.0%)	13 (31.0%)	3.6	1.5–8.7 **

## Data Availability

Data is available from the corresponding author upon reasonable request.
